# The Role of Oxidative Damage in the Pathogenesis and Progression of Alzheimer's Disease and Vascular Dementia

**DOI:** 10.1155/2015/504678

**Published:** 2015-08-02

**Authors:** Maria Luca, Antonina Luca, Carmela Calandra

**Affiliations:** ^1^Psychiatry Unit, Department of Medical and Surgical Sciences and Advanced Technologies, University Hospital Policlinico-Vittorio Emanuele, Santa Sofia Street 78, Catania, 95100 Sicily, Italy; ^2^Department of “G.F. Ingrassia”, University Hospital Policlinico-Vittorio Emanuele, Santa Sofia Street 78, Catania, 95100 Sicily, Italy

## Abstract

Oxidative stress (OS) has been demonstrated to be involved in the pathogenesis of the two major types of dementia: Alzheimer's disease (AD) and vascular dementia (VaD). Evidence of OS and OS-related damage in AD is largely reported in the literature. Moreover, OS is not only linked to VaD, but also to all its risk factors. Several researches have been conducted in order to investigate whether antioxidant therapy exerts a role in the prevention and treatment of AD and VaD. Another research field is that pertaining to the heat shock proteins (Hsp_s_), that has provided promising findings. However, the role of OS antioxidant defence system and more generally stress responses is very complex. Hence, research on this topic should be improved in order to reach further knowledge and discover new therapeutic strategies to face a disorder with such a high burden which is dementia.

## 1. Oxidative Stress and Brain Aging

Redox homeostasis is a complex mechanism that can be resumed as the maintenance of the balance between reactive oxygen species (ROS) production and elimination [1]. Largely generated from mitochondria, ROS are by-products of cellular metabolism [2]. Among them, we include free radicals (superoxide), hydroxyl radicals (the most reactive species), and nonradicals (hydrogen peroxide). Even though ROS exert a role in crucial physiological processes, such as signaling and apoptosis [3, 4], they are highly reactive species; as a result, they can damage proteins, lipids, deoxyribonucleic acid** (**DNA), and sugars with remarkable negative consequences on the cellular functioning [5]. The antioxidant defence system, composed by nonenzymatic and enzymatic antioxidants (e.g., glutathione, flavonoids, superoxide dismutase (SOD), catalases, and glutathione peroxidase (GPx)), protects the cells from the ROS-related injuries [6]. If the redox homeostasis fails, this system is not sufficient to counteract the high amount of ROS and the so-called “oxidative stress” (OS) occurs [7]. The balance between oxidants and antioxidants is not a static condition and a great number of stimuli can interfere with the redox status. Hence, OS has been recently redefined as a “disruption of redox signaling and control” [8]. OS and its detrimental effects on the cellular functioning have been demonstrated to be involved in aging [9], as well as in a variety of illnesses, particularly age-related ones, among which are diabetes [10], atherosclerosis [11], mild cognitive impairment (MCI) [12], Parkinson's disease [13], and other neurodegenerative disorders, such as Huntington's disease [14] and amyotrophic lateral sclerosis [15]. In addition, OS seems to be involved in the pathogenesis of the two major types of dementia: Alzheimer's disease (AD) and vascular dementia (VaD) [16]. The importance of OS in so many neurodegenerative disorders is not surprising, since the brain is highly susceptible to ROS, because

1. it is rich in fatty acids, which are sensible to peroxidation;
2. it has not a powerful antioxidant activity;
3. it consumes a lot of oxygen; therefore, it is exposed to free-radicals accumulation [17].

Previous researches highlighted the importance of OS in both normal brain aging and pathological brain aging [18], sharing the same altered biochemical and anatomopathological pattern: neural loss and altered mitochondrial activity and accumulation of degradated mitochondria and tangles [19–21]. However, AD brains show substantial qualitative and quantitative differences when compared to controls. More specifically, the redox homeostasis is different: the activity of mitochondrial pyruvate dehydrogenase, ketoglutarate dehydrogenase, and cytochrome oxidase is more severely affected; moreover, the antioxidant defence system is critically impaired [20, 22]. In addition, even though normal brain aging is related to the accumulation of degradated mitochondria and tangles, both conditions are more represented in AD [20, 21]. The amount of tangles certainly increases with age, particularly in the hippocampus. However, it is remarkably higher in demented brains and the more the dementia is severe, the more the tangles affect the neocortex, which is usually spared in normal brain aging [21].

High levels of peripheral markers of oxidative stress and low antioxidant power have been reported in patients with MCI, late onset AD, and VaD. Even though the three disorders seem to share a common oxidative-related pathogenesis, they maintain distinctive features, since other variables (e.g., homocysteine levels) allow for their differentiation [16]. An interesting study published in 2013 reported how young healthy individuals at risk of developing AD (as determined through genetic analysis) presented an apparently paradoxical condition: high levels of antioxidants and* reductive*, rather than oxidative, stress. On the contrary, in case of overt AD, the opposite situation was noticed: low antioxidants and high indicators of oxidative stress. The hypothesis is that the individuals at risk of developing AD, presenting an increased generation of ROS, respond with an overexpression of antioxidants, thus suffering from reductive stress. Later on, the antioxidant defence system collapses and the OS becomes evident, along with the symptoms of dementia [23]. The role of OS in the physiopathology of dementia is very complex and the knowledge pertaining to this topic needs to be enhanced. In the present review, data on the role of OS in AD and VaD, as well as a discussion on the therapeutic implications of such a role, are reported.

## 2. OS and AD

In the last decades, several researches investigating the role of OS in neurodegenerative disorders have been conducted. Despite the fact that the knowledge on this topic is certainly larger than before, it is still unclear whether OS is the cause or the consequence of the neurodegenerative processes. Notwithstanding, it is almost certain that OS is involved in the crucial events leading to the neural death and in the propagation of such events. Hence, if the complex and various neurodegenerative phenomena are intended as a cycle for more than a cascade, it is clear that, one way or another, OS is the “main actor” [24]. AD, the most common cause of dementia in the elderly, is an age-related neurodegenerative disorder causing the progressive loss of the higher cerebral functions, such as memory, language, and cognitive thinking, with huge consequences on mood, behaviour, and self-sufficiency [25]. This type of dementia is characterized by the accumulation of misfolded beta-amyloid (A*β*), a protein produced from the cleavage of the amyloid precursor protein (APP), and neurofibrillary tangles (aggregates of hyperphosphorylated tau protein) in the brain [26, 27]. Evidence of OS and OS-related damage in AD is largely reported in the literature [27–30]. More specifically, markers of lipid peroxidation have been found in plasma, urine, and cerebral tissue of transgenic mice models of AD amyloidosis [31]; OS-related DNA damage has been demonstrated in AD patients, so that some authors suggested that urinary oxidized nucleosides could be used as biomarkers [32]; high levels of carbonyls, markers of protein oxidation, have been found in AD brains [33]. Also tau phosphorylation has been related to OS and it is known that hyperphosphorylation is responsible for its misfolding [34, 35]. It is known that the ROS production is crucially involved in the physiological mechanisms regulating folding, misfolding, and the elimination of unfolded proteins [36–38]. The endoplasmic reticulum (ER) plays a fundamental role in the regulation of protein folding. In case of abundance of misfolded proteins, ER stress occurs, thus determining an enhanced production of ROS during the oxidative folding process (formation of disulfide bonds) and the uncontrolled accumulation of unfolded/misfolded proteins [36, 39]. The consequential depletion of the antioxidant glutathione and the ROS-related damage of the mitochondrial electron transfer system amplify the production of ROS and lead to cell death. In fact, the ER alteration is one of the common features linking neurodegenerative disorders, mostly characterized by protein misfolding, to each other [36]. In addition, a sustained OS alters the functions of the ubiquitin-proteasome pathway, which is responsible for the degradation of the damaged proteins [38]. As far as AD is concerned, there are several conditions causing the excessive production of ROS: mitochondrial dysfunctions, A*β*-related microglial activation, inflammation, and binding of redox active metals to deposits [40]. A deficiency in cytochrome c oxidase has been reported in platelets and in postmortem brain tissue of AD patients. As a result, mitochondrial degradation is stimulated and neurons are damaged by the mitochondrial debris and the excessive production of ROS [41]. As if that were not enough, the altered mitochondria generate high levels of ROS, exposing themselves to the OS-related injuries to which they are so sensitive [42]. Another important issue in AD is inflammation. In fact, this neurodegenerative disorder is characterized by an uncontrolled inflammatory activation of microglial cells [43]. The peroxisome proliferator-activated receptor gamma (PPAR-*γ*) is a regulator of the inflammatory processes which exerts anti-inflammatory properties [44]. OS, leading to the phosphorylation of PPAR-*γ*, is responsible for the functional alteration of this important transcription factor [45]. In addition, it has been reported that mild OS could trigger the amyloid cascade being, therefore, involved in the very early stages of AD: in fact, it causes an alteration of the subcellular compartmentalization of BACE1 (beta-site APP cleaving enzyme 1), an enzyme involved in the *β*-secretase cleavage of the APP; as a result, the amyloidogenic processing of APP is favoured [46]. As in a vicious cycle, A*β* produces ROS through a metal-catalyzed reaction [47]. The lesions observed in brains suffering from AD are those typical of OS (e.g., damage to DNA, protein oxidation, and lipid peroxidation) [18] and contain metals (e.g., zinc, iron) exerting catalytic activity and causing ROS production [48]; these metals have been demonstrated to be highly represented in AD brains [49]. From what has been discussed above, it is apparent that OS is involved in the occurrence of the core aspects of AD, that is, phosphorylation and misfolding [46, 50]. Obviously, the exposure of OS stimulates the activation of compensatory responses [51]. Unfortunately, both enzymatic and nonenzymatic antioxidant defences seem to be impaired in AD patients [52, 53]. Even though literature data are not exempted from inconsistencies [22], it is plausible that SOD could be induced by OS in the early stages of AD and, then, consumed in the advanced stages [22]. GPx and glutathione reductase (GR) seem to be, respectively, higher and lower in AD patients versus controls. Hence, the GR/GPx activity ratio turns out to be higher in healthy subjects, intermediate in MCI, and lower in AD patients. In addition, the ratio has been found to be positively correlated with the scores at the Mini Mental Sate Examination (MMSE), a tool used to assess the cognitive performances [12]. It is easy to imagine that since GR (which regenerates reduced glutathione from oxidized glutathione (GSSG)) is low, the levels of GSSG should be high. In fact, the levels of GSSG are higher in AD patients and relate to the severity of the dementia [22]. As far as nonenzymatic defences are concerned, even in this case literature data show some inconsistencies, but various studies report vitamin deficiency in patients versus controls [22]. Since the redox homeostasis is so deeply altered, the neurons are dangerously exposed to the detrimental effects of OS and to the fearsome mechanism of neurodegeneration.

## 3. OS and VaD

VaD is the second cause of dementia in the elderly. Executive functions, more than memory, are severely impaired in this type of dementia [54]. Even if hypoxia and haemorrhagic stroke (e.g., subdural haematoma) can cause VaD, the latter is mostly related to ischaemic stroke [55]. In particular, one of the most common forms of VaD, the subcortical one, is caused by multiple subcortical ischaemic lesions. Hypertension, diabetes mellitus, hypercholesterolemia, and hyperhomocysteinemia favour the occurrence of atherosclerosis, cardiovascular diseases, and stroke and represent important risk factors for VaD [54]. As far as oxidative stress is concerned, it is linked not only to VaD, but also to all its risk factors; in fact, OS has been demonstrated to play a role in the pathogenesis of diabetes [10] and to be involved in the tissue toxicity determined by hypercholesterolemia [56] and hyperhomocysteinemia [57]. Moreover, it has been reported that the dysfunction of mitochondrial proteins, leading to OS, is involved in the hypertension-related target organ damage affecting vasculature, heart, kidneys, and brain [58]. Mitochondrial dysfunction is considered to be an important step in the pathogenesis of atherosclerosis, also because it subtends the previously mentioned risk factors [59]. The OS-related oxidation of low-density lipoproteins (LDL) is crucial in the atherosclerotic process [60, 61] and high levels of lipid hydroperoxides have been reported in patients with ischemic stroke [62]. As a matter of fact, patients with VaD have been found to show high levels of malondialdehyde (a marker of lipid peroxidation) and these levels were higher than those reported in AD patients [63]. The association between folate deficiency and OS-related LDL dysfunction seems to be typical of VaD and could help to differentiate it from other types of dementia [64]. In addition, OS is certainly a mediator of the stroke-related neuronal damage and cognitive dysfunctions, as demonstrated by the high levels of plasmatic ROS in patients with ischaemic stroke as well as the finding of oxidative DNA damage within the peri-infarct brain regions in rats [65–67]. Moreover, OS is indirectly and directly involved in the deep alterations of the blood-brain barrier (BBB) occurring after an ischaemic brain injury. More specifically, the activation of metalloproteinases and the proteases involved in the proteolytic disruption of the BBB and in the white matter lesions typical of VaD is strictly linked to OS [68, 69]. In addition, ROS are directly responsible for the alterations in the cerebral perfusion and permeability, thus contributing to the cerebrovascular disease [70]. The OS-induced dysfunction of the previously cited anti-inflammatory agent PPAR-*γ* is involved in vascular aging [71]. In practice, OS and inflammation “cooperate” in determining the endothelial damage and the BBB failure occurring in VaD [72]. As in AD, even in VaD the antioxidant defence system seems to be insufficient. Apart from the previously reported folate deficiency [64], also vitamin E has been found to be lower in VaD versus controls and also versus AD [73]. In addition, SOD and GR are reduced in VaD too [74]. However, it is worth pondering over that, from a clinical point of view, the difference between AD and VaD is not so strict. In fact, microvascular degeneration and atherosclerotic processes are often documented in AD, in which an ROS-related amyloid cerebral angiopathy occurs [75–77]. In addition, the LDL oxidation, involved in the atherosclerotic process as previously mentioned [60, 61], seems to be a common feature shared by AD and VaD [78]. An important antioxidant enzymatic system, influenced by the redox status [79], protecting LDL from oxidation and exerting anti-inflammatory properties, is represented by the serum paraoxonases [78, 80]. Paraoxonase activity (as a protective factor) and macrophage OS (as a deleterious condition) take part in atherogenesis [81, 82]. Additionally, macrophage OS has been related to the paraoxonase 1 deficiency [83]. Considering dementia, both AD and VaD patients have been found to show a lower paraoxonase activity when compared to controls [78]. In the light of what has been discussed, OS represents a common important contributor in the pathogenesis of these two forms of dementia, whether alone or in comorbidity (mixed AD and VaD) [76]. Moreover, not only GR/GPx ratio, but also other markers of OS have been related to cognitive performances in dementia. High levels of 4-hydroxynonenal and malonaldehyde relate to worse scores at the MMSE [12, 84]. Notwithstanding, the use of biomarkers as predictors of severity or outcome of dementia seems to be not strongly enough supported, at least for now [22].

## 4. Antioxidants in AD and VaD: Future Therapeutic Perspectives

Several researches have been conducted in order to investigate whether antioxidant therapy exerts a role in the prevention and treatment of AD and VaD. The findings arisen from a cohort study considering more than 1300 subjects indicate that the intake of flavonoids is inversely related to the risk of incident dementia [82]. Hence, vitamin supplementation could play a positive effect in both AD and VaD [85, 86]. It has been demonstrated that curcumin reduces the levels of A*β* in cell lines and mouse primary cortical neurons and exerts a neuroprotective effect in vascular dementia enhancing the expression of antioxidants in rats and ischemic cells [87, 88]. In fact, the enhancement of the enzymatic defences is the key of the efficacy of treatments such as the EUK-207 (SOD/catalase mimetic) in mice with AD and resveratrol in rats with VaD [89, 90]. It has been reported that resveratrol increases SOD activity and glutathione levels in the cerebral cortex and hippocampus of rats with VaD [90]. Another research field is pertaining to the heat shock proteins (Hsp_s_), which has provided interesting findings. It is well known that OS is involved in the activation of such important chaperons, which regulate the aggregation of misfolded proteins and apoptosis [91]. However, in case of neurodegeneration, the chaperones could paradoxically facilitate the aggregation of disease-related proteins, attempting to repair them and trying to avoid the formation of toxic aggregates [92]. This data explains why Hsp90 inhibitors are one of the promising therapeutic tools for the treatment of AD [93, 94]. It has been affirmed that Hsp90 “may play a role in maintaining pathogenic changes that lead to neurodegenerative diseases” [95]. This concept is easier to understand if it is considered that the tau protein is a client protein for Hsp90 [96] and that the latter is a repressor of the heat shock factor-1 (HSF-1), which regulates the heat shock response through the expression of Hsp_s_ [93, 97]. Hsp90 inhibitors, through the activation of HSF-1, exert neuroprotective effects favouring the induction of Hsp_s_, such as Hsp70 [93, 98], that has been found to exert therapeutic properties in mice with AD [99]. Hsp_s_ are also expressed in response to ischemic brain lesions [100, 101] and transgenic mice expressing human inducible Hsp70 have shown to have ischemia-resistant hippocampal neurons [102]. Since inflammation plays an important role in the pathogenesis of AD and VaD [43, 72], the enhancement of the anti-inflammatory defence, through PPAR-*γ* agonists, could represent another potential target for the treatment of these severe dementias. In mice models of AD, PPAR-*γ* agonism resulted in the reduction of parenchymal A*β*, microglial activation, and neural loss [43]. In addition, it showed efficacy in improving reversal learning [103]. The oral antidiabetic drugs pioglitazone and rosiglitazone, exerting agonistic properties on PPAR-*γ*, were found to reverse some clinical (memory, learning) and biochemical (OS, endothelial dysfunction) features of diabetes-induced VaD dementia [104]. Moreover, their antiatherogenic effect is not only linked to their insulin sensitizing properties, but also to the modulation of endothelial activation markers, platelet activity, and vasodilatation. Hence, their therapeutic effects could be useful also for patients without diabetes mellitus affected by dementia [105]. However, the antioxidant therapy has not reached the aimed results. Among the more credible causes of this failure, (a) the activity of many nutritional antioxidants is strictly linked to that of other antioxidants; hence, monotherapy could not be sufficient; (b) therapy is often administered in too advanced stages of dementia; (c) since the brain is separated from the periphery trough the BBB, the peripheral redox status may not reflect the cerebral homeostasis; (d) the researches available used different methods to analyze the antioxidant levels in blood in order to evaluate the outcome after therapy [106]. Therefore, there are many fascinating plausible therapeutic targets that need to be further investigated to add new and more efficient therapeutic tools to the nowadays available disappointing options.

## 5. Concluding Remarks

In the light of what has been discussed, OS seems to be a crucial contributor in the pathogenesis of AD and VaD, directly or indirectly affecting the steps leading to neurodegeneration. AD and VaD are linked by many anatomical features, as well as by OS. However, the role of OS, antioxidant defence system and, more generally, stress responses, is very complex. Hence, research pertaining to this topic should be improved in order to reach further knowledge and discover new therapeutic strategies to face a disorder with such a high burden which is dementia.
